# Integration of Attributes from Non-Linear Characterization of Cardiovascular Time-Series for Prediction of Defibrillation Outcomes

**DOI:** 10.1371/journal.pone.0141313

**Published:** 2016-01-07

**Authors:** Sharad Shandilya, Michael C. Kurz, Kevin R. Ward, Kayvan Najarian

**Affiliations:** 1 Virginia Commonwealth University, Richmond, Virginia, United States of America; 2 Department of Emergency Medicine, University of Alabama School of Medicine, Birmingham, Alabama, United States of America; 3 Department of Computational Medicine and Bioinformatics, University of Michigan, Ann Arbor, Michigan, United States of America; 4 Department of Emergency Medicine, University of Michigan, Ann Arbor, Michigan, United States of America; 5 Michigan Center for Integrative Research in Critical Care, Department of Emergency Medicine, University of Michigan, Ann Arbor, Michigan, United States of America; University of Minnesota, UNITED STATES

## Abstract

**Objective:**

The timing of defibrillation is mostly at arbitrary intervals during cardio-pulmonary resuscitation (CPR), rather than during intervals when the out-of-hospital cardiac arrest (OOH-CA) patient is physiologically primed for successful countershock. Interruptions to CPR may negatively impact defibrillation success. Multiple defibrillations can be associated with decreased post-resuscitation myocardial function. We hypothesize that a more complete picture of the cardiovascular system can be gained through non-linear dynamics and integration of multiple physiologic measures from biomedical signals.

**Materials and Methods:**

Retrospective analysis of 153 anonymized OOH-CA patients who received at least one defibrillation for ventricular fibrillation (VF) was undertaken. A machine learning model, termed Multiple Domain Integrative (MDI) model, was developed to predict defibrillation success. We explore the rationale for non-linear dynamics and statistically validate heuristics involved in feature extraction for model development. Performance of MDI is then compared to the amplitude spectrum area (AMSA) technique.

**Results:**

358 defibrillations were evaluated (218 unsuccessful and 140 successful). Non-linear properties (Lyapunov exponent > 0) of the ECG signals indicate a *chaotic* nature and validate the use of novel non-linear dynamic methods for feature extraction. Classification using MDI yielded ROC-AUC of 83.2% and accuracy of 78.8%, for the model built with ECG data only. Utilizing 10-fold cross-validation, at 80% specificity level, MDI (74% sensitivity) outperformed AMSA (53.6% sensitivity). At 90% specificity level, MDI had 68.4% sensitivity while AMSA had 43.3% sensitivity. Integrating available end-tidal carbon dioxide features into MDI, for the available 48 defibrillations, boosted ROC-AUC to 93.8% and accuracy to 83.3% at 80% sensitivity.

**Conclusion:**

At clinically relevant sensitivity thresholds, the MDI provides improved performance as compared to AMSA, yielding fewer unsuccessful defibrillations. Addition of partial end-tidal carbon dioxide (PetCO_2_) signal improves accuracy and sensitivity of the MDI prediction model.

## Background and Significance

Sudden cardiac death remains one of the most challenging conditions to treat. In the United States, approximately 360,000 individuals suffer out of hospital cardiac arrest (OOH-CA) each year [1]. Despite the fact that a majority of these patients are treated by Emergency Medical Services (EMS) providers within minutes of collapse, survival to discharge remains dismal, varying regionally from 3% to greater than 16% [2]. Even though ventricular fibrillation (VF) is encountered in a minority of OOH-CA, it represents a significant, independent predictor of survival [1].

Since its first human use was described by Beck in 1947, defibrillation has been the accepted treatment for VF cardiac arrest [3]. VF is representative of a highly dynamic and deteriorating physiologic system. Typical quantitative analysis methods based on P, R and T waves cannot be applied to the VF Electrocardiogram (ECG), which depicts highly irregular morphology, changing periodicity and no recognizable P, Q, R, S, and T points. The timing of defibrillation has been controversial beyond the immediate cessation of coordinated mechanical cardiac activity in the setting of ongoing cardio-pulmonary resuscitation (CPR) [4]. Defibrillation attempts are generally timed at intervals that are arbitrary or defined by CPR algorithms *a-priori*, rather than at intervals defined by the physiological system’s current condition as optimized for success. Defibrillation when the OOH-CA patient is not physiologically “primed” for conversion to a perfusing rhythm can cause interruptions to CPR, which can subsequently impact countershock success in a negative manner [5–7]. In addition, it increases the number of unnecessary countershocks provided and cumulative electrical burden. Increases in the magnitude of electrical energy delivered are associated with decreased post-resuscitation myocardial function [8,9] and ultimately death.

Quantitative waveform measures (QVM) have demonstrated promise in differentiating response to defibrillation in animal models and retrospective human analyses [10]. Such QVM methods would potentially allow attending providers to rapidly predict shock success in real-time, reducing interruptions in CPR and defibrillation attempts with a low chance of success. Amplitude Spectrum Area (AMSA), which is a metric calculated from the frequency spectrum obtained by the Fourier Transform, is one such method that is currently commercially available but not in widespread use. AMSA may lack robust sensitivity and specificity because of severe limitations of the Fourier Transform in characterizing non-stationary biomedical signals [10]. Independent studies have found significant overlap of AMSA values within a single standard deviation of the mean among survivors and non-survivors [11,12]. While statistically significant, more robust computational testing of the AMSA measure may prove it to be a weak discriminator for decision support.

Different signal processing techniques that are capable of taking advantage of both frequency and time elements of signals coupled with advanced computational artificial intelligence (machine learning) techniques may offer advantages in developing more robust decision tools where the biologic signal and physiologic process under study are likely to be highly nonlinear in nature. The goal of this investigation was to develop a unique real-time machine learning (ML) method using multiple QVM and signals to predict VF defibrillation success, evaluate the underlying quantitative assumptions, and compare its performance in context of other available technologies. The methods would form the basis of a technology that delivers recommendations to the interventionist in real-time, utilizing information from ECG segments of short duration.

## Materials and Methods

### Study Design

The study was a retrospective analysis of anonymized cardiac arrest data including continuous ECG and partial end-tidal carbon dioxide (PetCO_2_) measurements and electronic medical records generated by pre-hospital providers. This investigation was approved by the Institutional Review Board at Virginia Commonwealth University in Richmond, Virginia.

Data for 153 out-of-hospital cardiac arrest (OOH-CA) patients whose resuscitation involved a period of ventricular fibrillation (VF) for which they received at least one attempt at conversion to a perfusing rhythm via defibrillation was provided by the Richmond Ambulance Authority (Richmond, VA) and Zoll Medical Corp. (Chelmsford, MA). Any other individually identifiable data was removed to prevent direct or indirect linkage to specific individuals by the investigators. Prior to computational analysis, shocks were manually confirmed and classified as either successful or unsuccessful by both an emergent cardiac care specialist M.C.K. (coauthor) and by an emergency medicine specialist K.R.W. (coauthor), based on the post-defibrillation ECG segments and data from the pre-hospital care record. Successful defibrillation was defined as a period of greater than 15 seconds with narrow QRS complexes under 150 beats per minute with confirmatory evidence that return of spontaneous circulation (ROSC) had occurred. Such evidence included lack of CPR resumption over next minute, mention of ROSC in the electronic record, and/or rapid elevation in PetCO_2_ levels. A total of 358 countershocks were deemed usable for analysis (218 unsuccessful and 140 successful).

Python was used for parsing and manipulating data, Matlab^®^ software was used for signal processing, and open source Weka^®^ [13] was used for machine learning.

### Pre-Processing

Signals were filtered by utilizing an adaptive method [14] as follows. The method is geared toward preserving high-frequency end of the signal while focusing on significant baseline drifts.

Step 1: Reduce high frequency noise using Savitzky-Golay low-pass (smoothing) filter. 11^th^ degree polynomials are fitted to frames of 25 samples.
Step 2: De-Trending
  > Step 2a: Successively smooth the signal until only baseline shifts and drifts, caused by noise and interference, remain. 3^rd^ degree polynomials are fitted to frames of 499 samples or less. The number of samples must be odd.
  > Step 2b: Subtract the new signal (from step 2) from the signal (from step 1)

**Fig 1:**
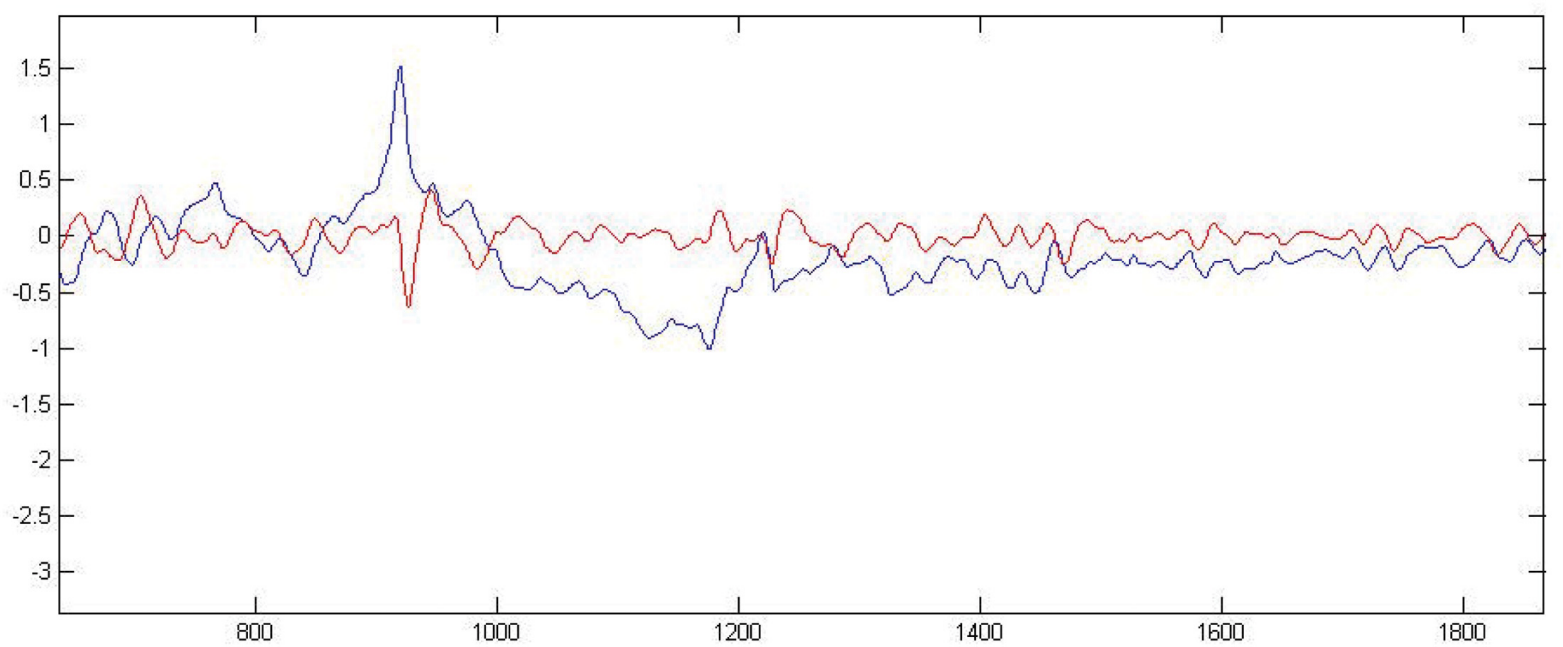
Filtering. Blue: Original signal with a sudden jump around sample 900 and then a drift till sample 1200. Red: Filtered signal displaying physiologic morphology around sample 900 and no drift till sample 1200. y-axis::mV, x-axis::samples.

Raw and filtered signals are plotted in Fig 1. Filter parameters have remained unchanged and adequate since their first use on a much smaller dataset reported in 2011 [14]. A supervised dataset was built with 9 second pre-countershock signal segments and corresponding outcomes.

### Testing the Basis for Non-Linear Dynamic Methods

Decomposing a short-term/non-stationary, pathological system requires assumptions of linearity and periodicity, such as that of the FT, to be relaxed. Limitations of a Fourier based analysis have also been discussed in other studies [15,16]. The Quasi Period Density—Prototype Distance (QPD-PD) method is based on non-linear time-series analysis, which helps in bridging the gap between deterministic chaos theory [16, 17] and observed “randomness” of a system. Methods of non-linear time-series analysis arise from the theory of deterministic dynamical systems [16]. The ‘embedding’ theorem [18,19] can be used to construct a multidimensional phase space from a single variable. Dimensions of the phase space P correspond to multiples of the delay *τ*.

$$P=\left[p_{n},p_{n-\tau },\ldots ,p_{n-\left(m-1\right)\tau }\right]$$ (1)

The value of each dimension (from Eq 1) at time *t* corresponds to the value of the signal at times: *t = i Δt*, *t* = (*i*+*τ) Δt*,…, *t =* {*i+*(*m*-1)*τ*}*Δt*, where *i* is the sample index. Here *Δt* serves as an operator and represents the time between each sample, i.e. (*sampling rate*)^-1^ of the signal. For a fixed *m* (optimized at 4 dimensions for the given dataset),

1. *τ* has to be large enough so that the information at *i+τ* is significantly different from the information at *i*. Once a proper *τ* (optimized at 8 samples for the given dataset) is chosen it will give us enough information to construct the phase space.
2. On the other hand, the system may appear not to have any memory if *τ* is chosen to be too large.

Based on the optimized parameters, QPD is constructed for each signal. Depending on the actual amount of information (about the system) present in the signal segment (which may partly be a function of the length of the segment), ‘loss of memory’ is also a characteristic of chaotic systems, where a small change in initial conditions produces a large divergence in trajectory in the phase space. It is important to note that the effect of incomplete information about a complex dynamic system (such as the cardiac system in arrest) may produce properties that are similar to that of a chaotic system. In both cases, the system will appear to lose the memory of its initial state and may therefore become unpredictable in time. The Lyapunov exponent [20] quantifies the rate of divergence of two trajectories in the phase space, and would serve to form rationale for non-linear methods used. If the initial separation of two trajectories is given by *ΔS*_0_, they diverge according to the rule

$$|\Delta S\left(t\right)|=e^{\lambda T}\cdot \left|\Delta S_{0}\right|$$ (2)

For a discrete time system, where *S*_0_ is the starting point of the orbit, and *S*(*t*+1) is a function of *S*(*t*), the Lyapunov exponent can be expressed as

$$\lambda =\;{\text{lim}}_{n\to \infty }\left(\frac{1}{n}\sum_{i\;=\;0}^{n-1}\text{ ln}\left|\frac{dx_{i+1}}{dx_{i}}\right|\right)$$ (3)

**Fig 2:**
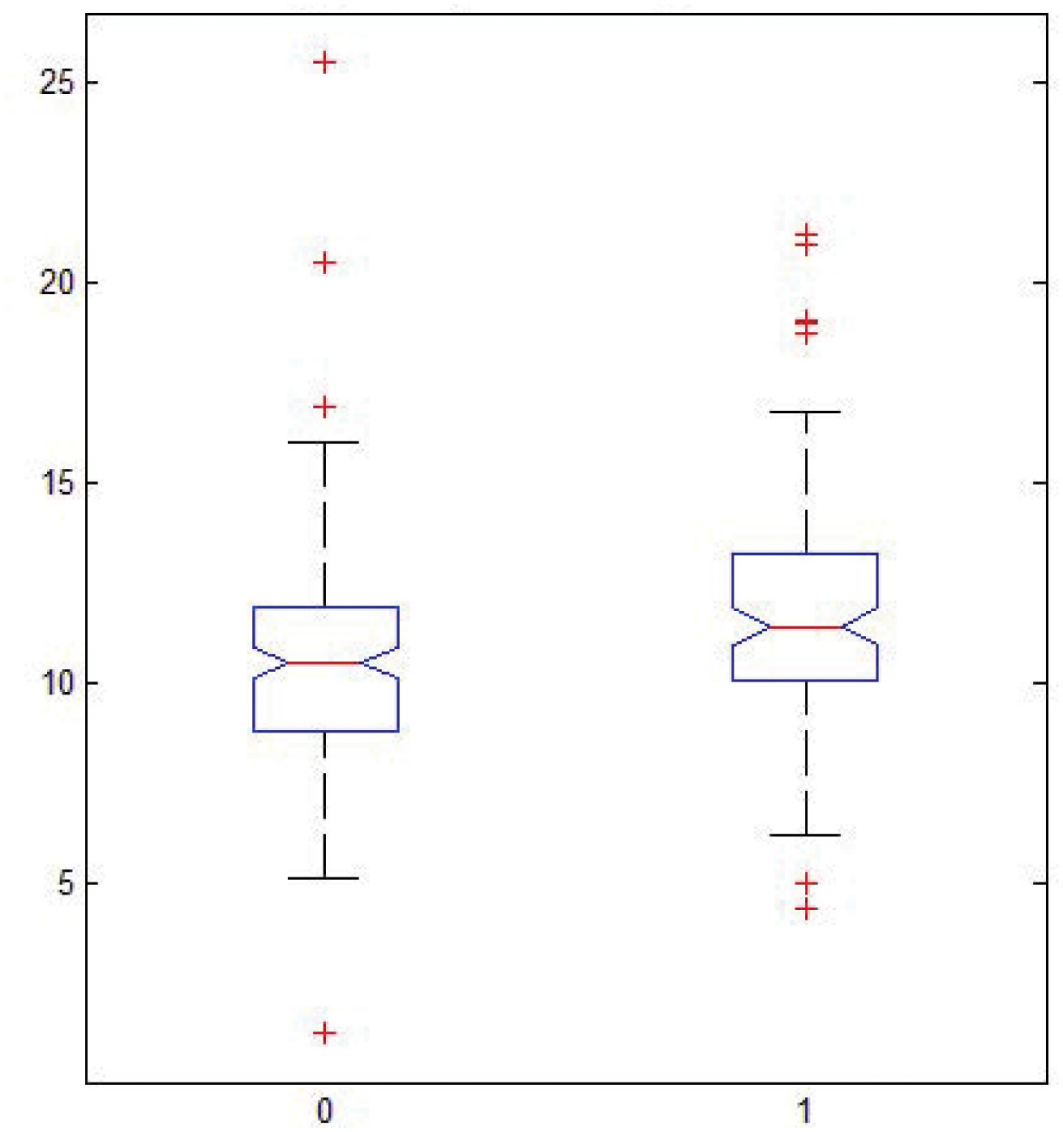
Maximal Lyapunov Exponent of VF. Two boxplots, one for each class, representing distributions of maximal Lyapunov exponent (y-axis) for all signals. x-axis: "0" signifies "unsuccessful" class, while "1" signifies "successful" class.

**Fig 3:**
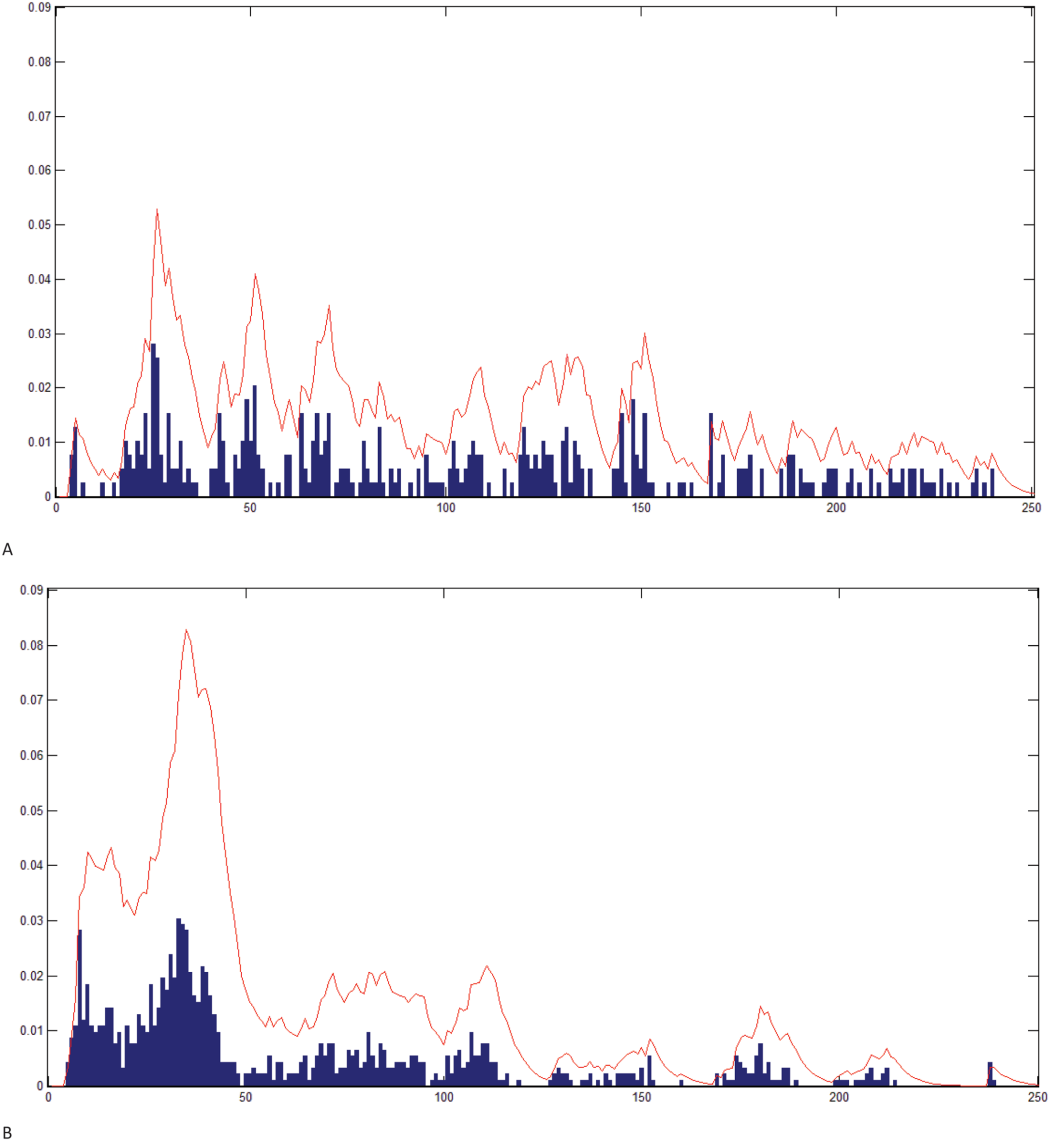
Quasi-Period Density Function. QPD for (A) a successful shock and (B) an unsuccessful shock. Bars represent the normalized amplitude for each pseudo period: The line curve on top of the histogram represents QPD convolved with the exponential function. If most of the Quasi-Periods are clustered within a small subset of values, as is (B), the convolution helps quantify that fact.

A positive Lyapunov exponent (Fig 2) indicates that the underlying system is chaotic. Quantification of Lyapunov exponents show that the limited duration VF ECG segments exhibit chaotic non-linear dynamic characteristics. Additionally, topological mixing is a necessary property of a chaotic system [21], but proving this property is not necessary for our proposed model. The quasi-period plots (Fig 3) can represent deterministic/stochastic, non-dynamical/dynamical, stable/unstable (chaotic) properties of a system.

Contrastingly, Fourier transform (FT) [22] performs a linear transformation of a function space such that the original signal (function) is decomposed into multiple sinusoids. A tradeoff exists between signal length and frequency resolution. In other words, for a given fixed-duration segment, the Fourier basis is not localized in space/time. Previous studies have *not* utilized the above mentioned non-linear dynamic methods for the purpose of predicting defibrillation outcomes. Since QPD’s have a non-linear non-deterministic basis for characterization of ECG’s, the features extracted from them are hypothesized to be strong predictors. This hypothesis is proven through statistical testing as well as the relatively strong discriminative performance of the MDI model as compared to the leading method AMSA.

### Feature Extraction and Statistical Analyses

Decomposition and non-linear methods enable us to define and extract characteristics (features) of a system that may be predictive of the outcomes (success/failure of a shock delivered) and can be used to induct a machine learning model that is predictive of such outcomes. Wavelet Transform (WT) based methods [12] augmented by a dual-tree decomposition algorithm [22] were used to overcome limitations inherent to FT based methods [23] and eliminate shift-variance, which leads to large changes in wavelet coefficients due to small shifts in the signal. Since the signal segments are extracted by windowing, the latter presents a significant problem.

Quasi Period Density—Prototype Distance (QPD-PD): The previously described QPD-PD method [24] was used to characterize chaotic signals from their phase space while allowing for stochasticity/non-determinism. The method's focal point is the Probability Density Function (PDF) of the quasi-period. As illustrated in Fig 3, the PDF is calculated by convolving the quasi-period density with the exponential function (Eq 4 below). The PDF helps quantify the difference in densities between the two classes, 'successful' versus 'unsuccessful'. In the following convolution, *q* is the quasi period density and *exp* represents *e*^-t/4^.

$$\left[exp*q\right]\left(t\right)=\;\int_{0}^{+\infty }\text{exp}\left(\tau \right)q\left(t-\tau \right)d\tau$$ (4)

QPD-PD’s parameter selection and feature calculation are geared for discrimination between classes. Four post-defibrillation signals exhibiting regular sustaining sinus rhythms, with narrow complexes, were used to select the corresponding pre-defibrillation signals as successful prototypes. Similarly, signals preceding four countershocks that induced minimal change in the ECG or were immediately followed by smooth VF, with no conversion, were selected as unsuccessful prototype signals. The resulting set of (8) pre-countershock signals is termed the Prototype Set (PS). The quantity *sep*, defined below, is then utilized as the maximization criterion for selection of QPD-PD’s parameters by discriminating successful prototypes from unsuccessful prototypes.

$$sep=\;\overset{L}{\underset{i}{\sum }}\frac{\bar{KD_{i}^{B}}\;-\;\bar{KD_{i}^{W}}}{\text{max}\left(\;\sqrt{\frac{1}{C^{B}}\sum_{j=1}^{C^{B}}{\left(KD_{i}^{j}-\;\bar{KD_{i}^{B}}\right)}^{2}},\;\;\;\sqrt{\frac{1}{C^{W}}\sum_{j=1}^{C^{W}}{\left(KD_{i}^{j}-\;\bar{KD_{i}^{W}}\right)}^{2}}\right)}$$ (5)

Here, *L* is total number of signals from both classes in PS. For a given signal *i*, *KD*^*B*^ and *KD*^*W*^ in the numerator are means of distances from PS signals in opposite-class and within-own-class, respectively. *C*^*B*^ is the total number of prototype signals in the opposite class while *C*^*W*^ is one less than the number of prototype signals in *i*’s own class. The distance measure *KD* is calculated by comparing the PDFs of quasi-periods [12]. *KD* represents the distance of the given signal’s QPD from the QPD of a signal in the prototype set. *sep* serves to separate the signals in ‘successful’ PS as far as possible from signals in ‘unsuccessful’ PS [12]. While *KD* is used for parameter selection, *sep* can be used as a general discriminant heuristic that does not necessarily need to be defined in terms of *KD*.

**Fig 4:**
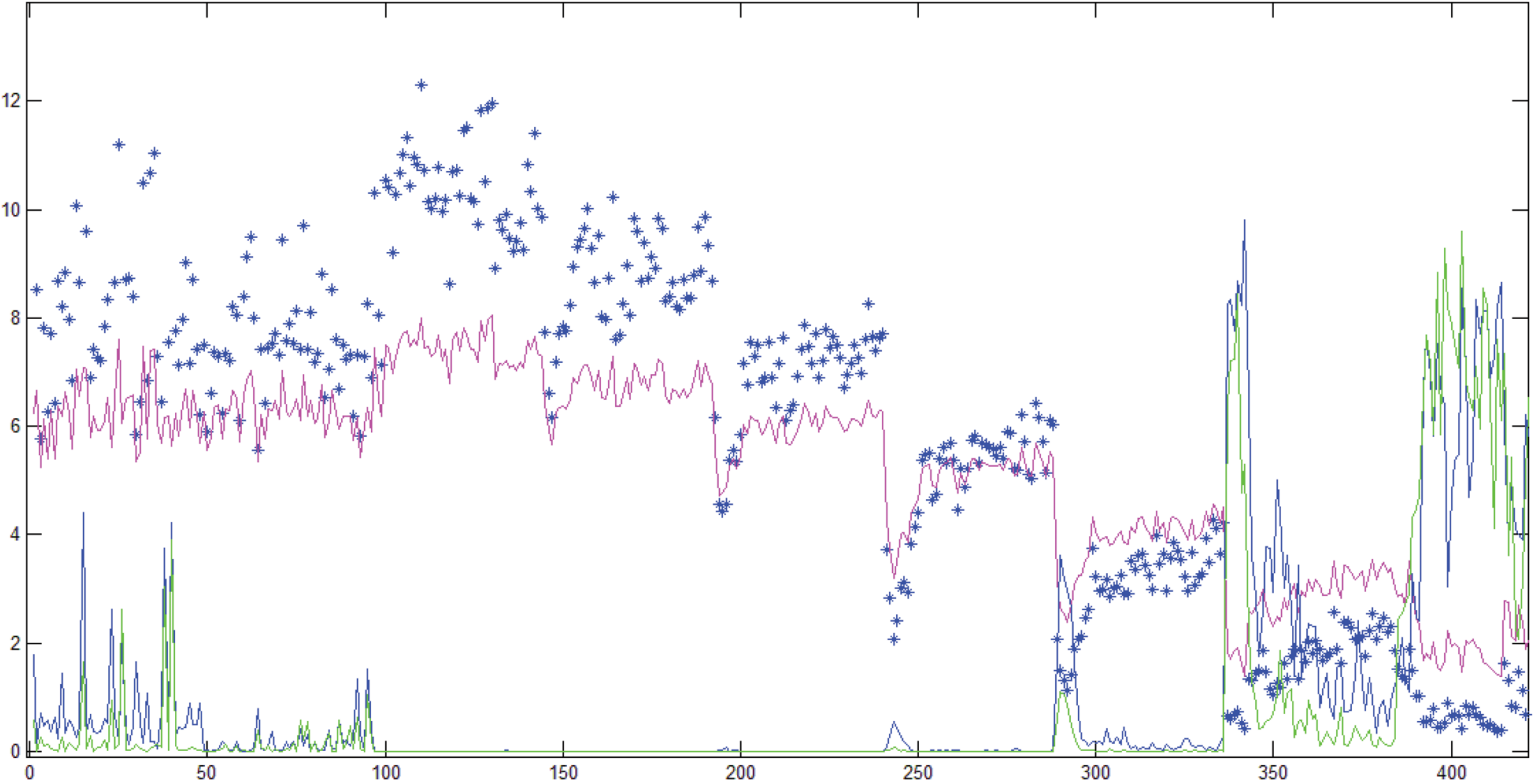
Heuristics and Test Statistics. X-axis: Different combinations of parameter values for the QPD-PD method. Y-axis: Scaled Probability of False Positive (for Blue and Green lines) or Values of Measure (for Blue Stars and Pink Line). Blue Stars: *F* measure, Pink Line: *Sep* measure, Blue Line: ANOVA Probability of False Positive, Green Line: Kruskal-Wallis Probability of False Positive.

*Sep* (Eq 5) is also utilized to calculate the final set of extracted features or explanatory variables. Each scalar value of a feature is representative of one signal segment. We compare *sep* with other traditional, well-established parametric and non-parametric heuristic and hypothesis tests, namely the *F* statistic, analysis of variance (ANOVA) and Mann-Whitney-Wilcoxon (MWW) rank-sum test (Fig 4).

With each unique combination of parameter values for a QPD representation of the signal, one feature-set is constructed. The outcome variable (class) is appended to the vector of features (explanatory variables) representing a snapshot of the cardiovascular system preceding each countershock. Explanatory variables serve as input to a trained ML model (MDI) which then classifies the corresponding instance to a given class (prediction). To facilitate hypothesis testing, the relationship between outcome and explanatory variables was inverted. Specifically, the class variable can be considered a treatment with two factor levels, successful versus unsuccessful, while each explanatory variable would be a measured response.

As the number of features (equivalently, the feature space dimensionality) grows, chances of finding variables that spuriously correlate to outcomes for the given (finite) sample set also grow. This leads to overfitting while training, potentially yielding a seemingly high-performing (on sample set) machine learning model [25]. Additionally, feature and parameter selection on a large number of features become sub-optimal or computationally infeasible [26]. The following processes and techniques undertaken during the study tackle problems associated with high dimensionality:

- Statistical validation of features through parametric and non-parametric methods, as well as through *multivariate* analysis of variance.
- Dimensionality reduction
- Parameter selection and feature selection within a nested cross-validation setup

ANOVA and Kruskal Wallis (KW) test were used to evaluate the significance of each feature with respect to the treatment. The null hypothesis states that class (outcome) is not associated with different pre-countershock cardiac states as represented by each feature value. Repeating ANOVA and KW test for each feature aids in comparison of *sep*, *F* statistic, and KW test. ANOVA was carried out for all the features, since it is well-known to be a robust method even in cases where normality is not satisfied. The problem of accumulation of probability of false positives because of repeated testing is dealt with a Multivariate Analysis of Variance (see next section). Notably, for the two class case, ANOVA reduces to a T-test. In Fig 4 titled “Heuristics and Test Statistics”, the *F* measure has been plotted in the same color as the ANOVA curve to reflect the fact that the probability of false positives for ANOVA is calculated from the value of the *F* measure and is therefore directly proportional to it. An arbitrary significance level was not fixed apriori. Each point on the plot corresponds to the total probability of false positives (Y-axis) accumulated with tests conducted for each set of 40 features from the feature set. The probabilities were scaled up by a factor of 10 for visualization on the same plot with *sep* and *F*.

ANOVA assumes a normal distribution for a feature with respect to each class, while KW test is the non-parametric equivalent of ANOVA. KW test can therefore assess features that are non-normally distributed with respect to each factor level (class). Additionally, KW test may serve to be more conservative than ANOVA since our design is not balanced, i.e. class memberships are imbalanced (218 unsuccessful versus 140 successful). Some loss of information is incurred because continuous feature values are converted to ranks. For the two class case, KW test amounts to a MWW rank-sum test. About 20% of the features extracted showed non-normal skewed histograms for both groups. ANOVA yielded a larger probability of false positives *P*(*fp*) where KW test also showed an increased *P*(*fp*) for the corresponding QPD. Each QPD representation corresponds to one unique combination of parameter values. KW test resulted in a higher *P*(*fp*) than ANOVA for very few QPD representations (Fig 4), while ANOVA yielded higher P(*fp*) otherwise. *Sep* and *F* measures agree with each other for all cases, while *sep* shows a greater amount of proportional variance (variance normalized by the mean value) as compared to *F*. Both *F* and *Sep* measures show a relatively high variance for models 0 through 50. In contrast, for models 300 through 330, the heuristics show smaller variance but also a smaller mean value. Yet, the first 50 representations yield features that lead to large P(*fp*), even though the values of the heuristics are relatively large. Therefore, increased relative variance within a ‘neighborhood’ of parameter values may be indicative of spuriously inducted models. This indication is being explored further in a separate study.

**Fig 5:**
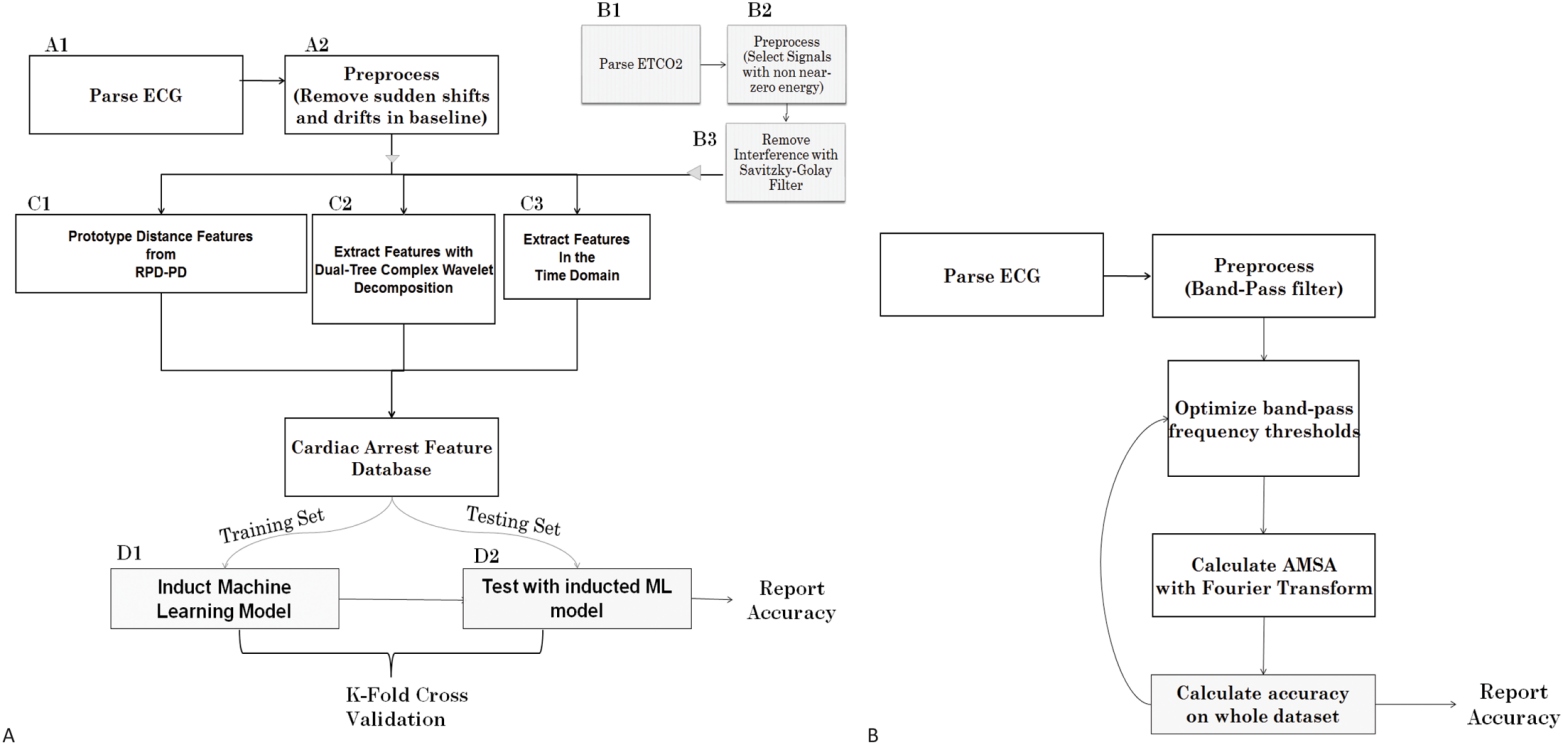
**A. Overview of the MDI system.** Components labeled A and B represent pre-processing and filtering. C represent non-linear modeling, decomposition, and feature extraction. D represent machine learning model induction and testing. Additional statistical analyses such as KW test and MANOVA were performed with the feature database created by C. **B. AMSA feature/method.** Flowchart represents the sequence of steps involved in the AMSA method, with the two major methodological components being the filtering (low-pass and band-pass) and the Fourier Transform.

Dual-tree complex wavelet transform and other time-series features were also calculated and incorporated into the feature set [14]. An overview of the system is displayed in Fig 5A. QPD-PD and Wavelet-based decomposition constitute data characterization and feature/information extraction components of the overall MDI system (Fig 5). The final machine learning model that is capable of performing predictions is termed the MDI model here.

### Dimensionality Reduction

Projecting the feature space onto a new set of orthogonal axes *Z* is a common technique utilized in many fields ranging from social sciences to microbiology. The technique is used with the hope that the first few dimensions of the new coordinate space *Z* will represent a large majority of the total variance, and that the rest of the dimensions/features can be discarded by making the assumption that the variance represented in them is spurious [25].

The feature set, consisting of distances calculated with QPD-PD, various statistical properties of the wavelet coefficients, and time-series features [12], was first projected onto a new orthogonal set. Each new dimension has a corresponding eigenvalue that quantifies the proportion of total variance in the feature set covered by that dimension [25]. Starting from the new feature with the largest eigenvalue and continuing till a cumulative variance close to 99% was reached, the rest (about 40%) of the features from the new set could be discarded [12]. As such, by discarding about 1% of the total variance, a significant reduction in dimensionality was achieved. This makes the subsequent task of feature selection significantly more optimal as well as computationally feasible (data provided in S1 Data).

Prior to dimensionality reduction/orthogonalization, ANOVA served to test each feature with respect to outcomes. Multivariate Analysis of Variance (MANOVA) on the now orthogonal feature set provides a holistic answer to the question: ‘Is the extracted feature-set significantly different across classes?’. MANOVA can be seen as an extension of ANOVA for multiple dependent variables that are preferably uncorrelated, since collinearity can lead to unstable estimate of discriminant function coefficients and an increasing number of (correlated) responses results in loss of degrees of freedom, thereby limiting benefit. Additionally, reduced dimensionality results in increased robustness to heterogenous variance-covariance matrix. In order to conduct MANOVA, dimensions from the uncorrelated feature set were treated as responses and the class was treated as a factor. Notably, MANOVA for two factor levels reduces to a multivariate T-squared test.

### Comparing ML Paradigms and Algorithms

Inductive ML algorithms can create a mathematically expressible function, as demonstrated in numerous decision rules in medicine derived from logistic regression [26]. For example, a number of risk scores, such as TIMI, have been developed for predicting the risk for cardiovascular complications [27]. The ‘No Free Lunch’ theorem [28] establishes that no specific algorithm can be guaranteed to provide the highest performing model for a given finite dataset. Multiple ML methods, including back-propagating neural networks,^19^ Random Forest Tree Induction [29], and traditional Bayesian logistic regression [30] were utilized to induct models with the supervised feature sets. We selected algorithms that are well-known in the field of machine learning, have been researched thoroughly for several years. All performance metrics for the inducted MDI models are presented in Table 1.

**Table 1:** Results of each compared Machine Learning Approach.

ML Approach	Accuracy	ROC-Area	Optimized Parameter Values
Random Forest	75.1	79.9	Trees = 100, % of features = 80
Bayesian Logistic Regression	78.8	76.8	Gaussian Prior (versus Laplace Prior)
Back-propagation NN	77.4	83.7	Iterations = 500; Learning Rate = 0.3; Momentum = 0.4
Adaboost C4.5 Trees	78.2	78.4	Iterations = 100

Receiver Operating Characteristic (ROC) analysis was used to evaluate reliability of all models by calculating the area under the curve (AUC). Accuracy was calculated as the average percentage, over all cross-validation runs, of instances correctly classified. All *accuracy*, *sensitivity* and *specificity* values are reported for the best decision threshold found for the given test and/or algorithm. These statistical measures are reported at both 80% and 90% sensitivity levels. Conservative nested ten-fold cross-validation, in which parameters are selected inside the training sets to avoid overfitting, was used for all tests so as to obtain an unbiased estimate of accuracy, sensitivity and specificity. In this validation process, data is randomly divided into ten partitions (folds). During each step of validation process (i.e. each outside loop), a combination of nine partitions is used for training the MDI model while the last partition is used for testing the trained model. This process is repeated ten times, each time using a previously unused fold for testing.

**Fig 6:**
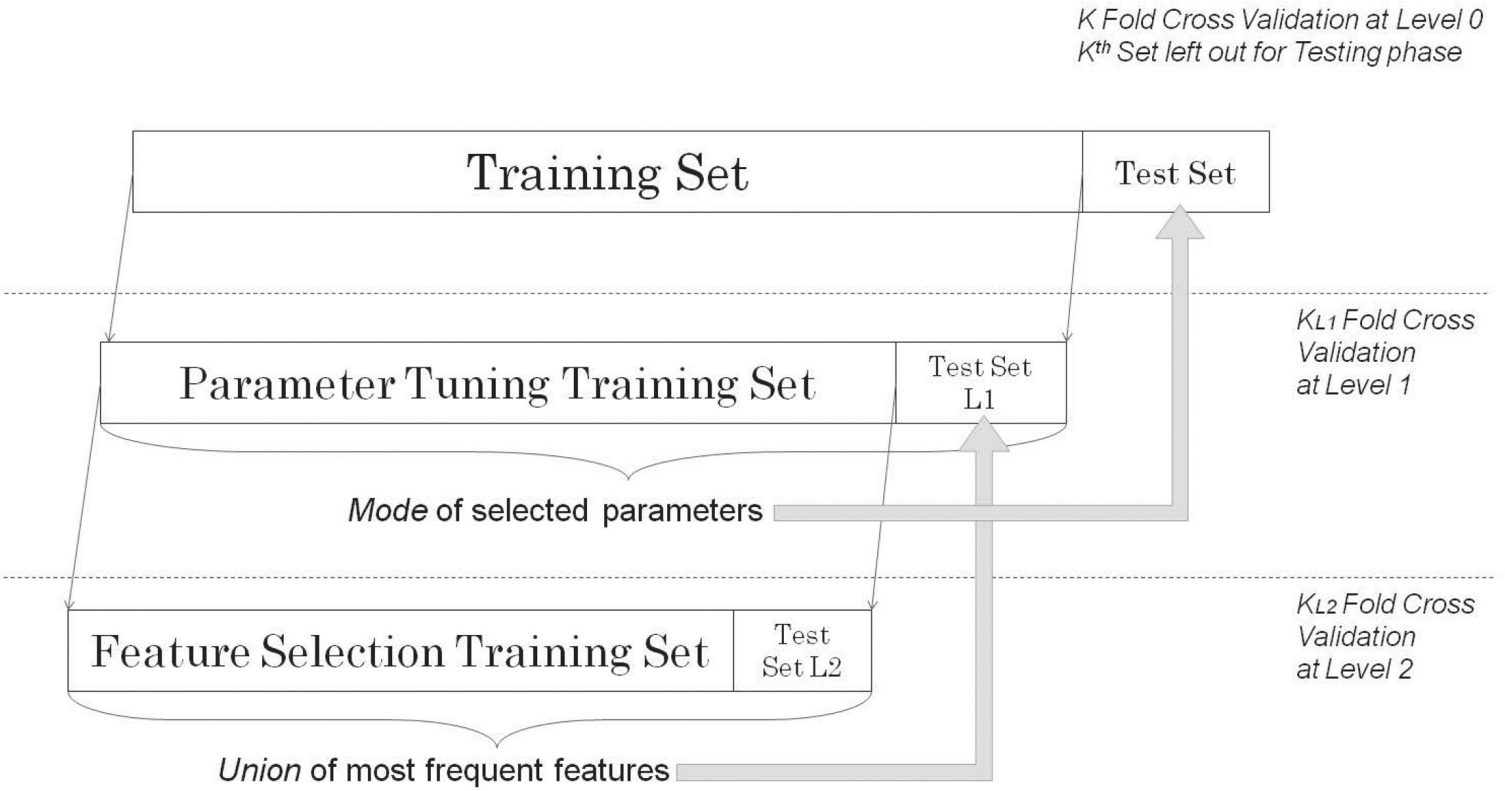
Framework for Wrapper Based Hyper-Parameter Selection. Twice-nested cross-validation setup. Parameter tuning is performed at Level 1 (L1), where an optimal feature subset has already been selected by cross-validation at Level 2 (L2). k = k_L1_ = k_L2_ = 10 folds; same for all levels.

In nested architecture (Fig 6), for each outside loop, a subset of data inside the combined nine training sets is used for selection of features for the model. As such, the selected features vary for each outermost test fold, while the global set of features (as well as feature extraction and selection algorithms) stay constant. This feature selection process significantly reduces the chances of overfitting (positive bias on reported accuracy) with respect to parameter selection process [11]. In contrast, the AMSA (Fig 5B) method does not employ cross-validation or nested cross validation in order to select parameters (such as frequency sub-band, filtering threshold) or to estimate performance metrics.

## Results and Discussion

**Fig 7:**
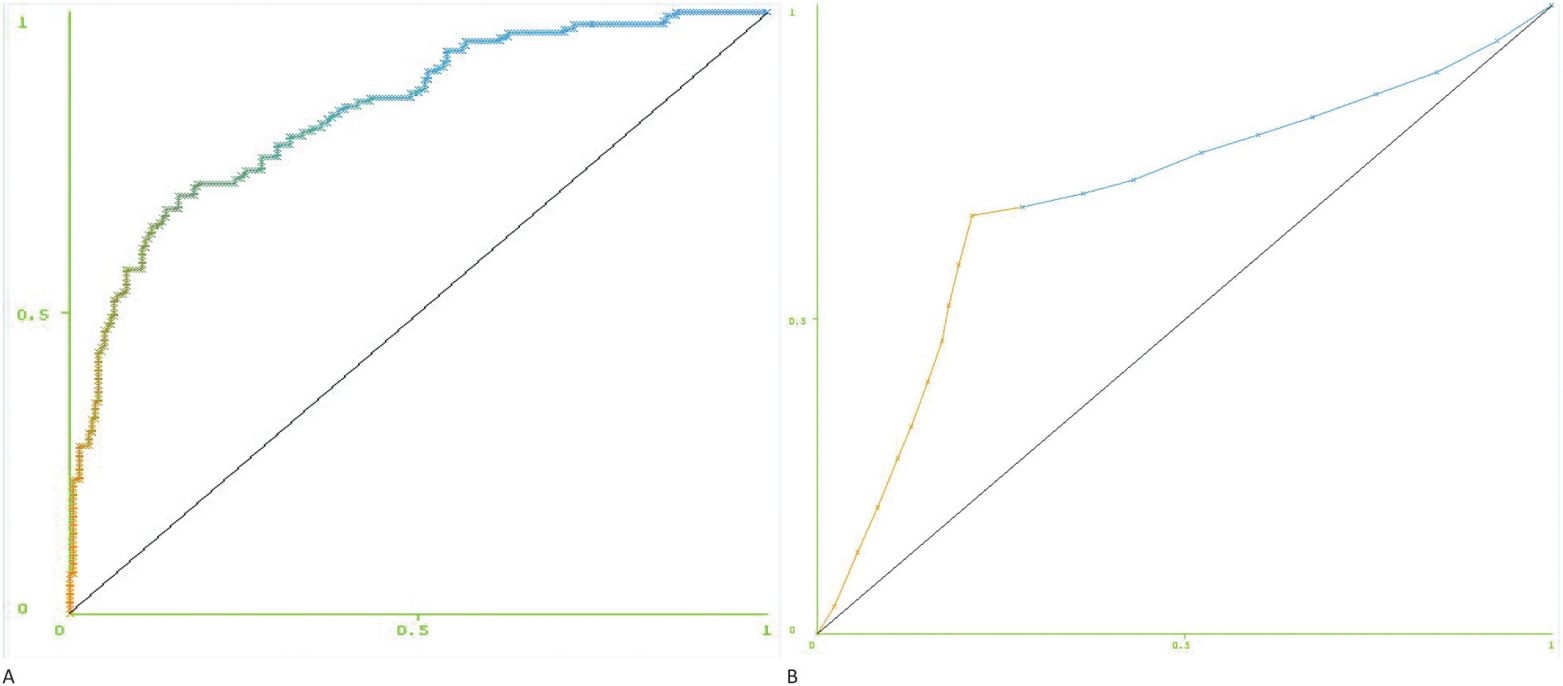
Receiver Operating Characteristic curves. For (A) MDI model built using all 358 shocks, (B) AMSA method. X-axis = 1-Specificity, Y-axis = Sensitivity. Threshold ranges from 0 to 1 as the color transitions from orange to blue from one end to the other.

Classification using MDI with additive logistic regression [31] as a classifier, with up to 20 features, yielded an ROC AUC of 83.2% for the model built with ECG data only (Fig 7A). Multiple comparisons of MDI and previously reported AMSA method [23] were performed. AMSA yielded an ROC AUC of 69.2% (Fig 7B).

Specificity can be calculated at desired levels of sensitivity by adjusting the decision threshold of a classifier. If the classifier’s output is continuous, this threshold can be set anywhere within the range of the output. For logistic regression, continuous values between 0 and 1 represent the probability of a successful shock according to the model. At 80% sensitivity (threshold of .41), MDI provided an accuracy of 74% and specificity of 70.2%. At the same level of sensitivity (80%), AMSA provided an accuracy of 53.6% and specificity of 36.7%. While increasing that sensitivity to 90% (threshold of .22) yielded an accuracy of 68.4% and specificity of 54.6% with MDI, performance of AMSA dropped dramatically to 43.3% and 13.3% respectively (Table 2).

**Table 2:** Performance of the MDI model in comparison with AMSA.

Accuracy	Proposed Model	AMSA Feature
Overall	78.8%	73.9%
80% Sensitivity	74%	53.6%
90% Sensitivity	68.4%	43.3%
ROC-Area	83.2%	69.2%

Integrating PetCO2 features into MDI boosted ROC AUC to 93.8% for a total of 48 shocks with usable CO2 signal segments. At 90% sensitivity, the large ROC AUC allowed for 83.3% accuracy and 78.6% specificity.

MANOVA was carried out on the resulting orthogonal (uncorrelated) feature set, yielding (*p*<0.05), and thus rejecting the null hypothesis that the class (outcome) is not associated with different pre-countershock cardiac states as represented by the set of features.

Predicting the success of defibrillation would minimize interruptions in CPR and unnecessary shocks, both of which can reduce chances of ROSC and ultimate survival to discharge. Physiologic changes during CPR take place over short intervals as the compressions and pharmacotherapy attempt to improve myocardial perfusion. In the study presented, the MDI model was able to discriminate with high accuracy those defibrillations that effectively converted VF to a perfusing rhythm and those that did not. Predictions are computed in real-time (<.08 second delay per prediction) and are based on information gathered from signal segments 9 seconds in duration.

A few predictors of successful resuscitation exist. These include physiologic parameters such as coronary perfusion pressure (CPP) [31], central venous oxygen saturation (Scvo_2_) [32], PetCO_2_ [33, 34], and QVM of the ECG waveform [35, 36]. While directly correlated to cardiac output and highly sensitive for ROSC, CPP and Scvo_2_ are mostly impractical to measure during cardiac arrest outside of the intensive care unit (ICU) setting. Waveform capnography to measure PetCO_2_ is practical in most settings, including the pre-hospital environment, and is highly correlated with CPP [37], cerebral perfusion pressure [38], and ROSC [39,40]. However, its ability to predict defibrillation success has not been established.

Without an ideal monitoring technique to predict the success of defibrillation that is practical in all cardiac arrest settings, QVM has emerged as a technology that can be integrated into existing defibrillator units. The most prominent, AMSA, relies upon a single feature of VF derived from the Fourier Transform to predict defibrillation success. In certain animal [41,42] and human [23,43] investigations, AMSA has been shown to predict defibrillation success with greater than 90% sensitivity and specificity. However, these studies did not employ cross-validation in their analyses, which yields an unbiased and significantly more conservative estimate of performance for unseen data while utilizing the entire dataset available. Although statistically significant, generalization performance of AMSA as a discriminator has been found to be much lower in recent studies [11]. The feature and parameter selection framework for MDI allows us to judge the success of current research efforts and to provide the right foundation for potential translational research.

In the field of artificial intelligence, the technique of ML is capable of utilizing numerous features extracted from a signal(s) to identify significant patterns, which match a classification of interest. In this case the classification of successful versus unsuccessful defibrillation is used. Each one of the features can contain information that is complementary to the information present in other features. All such discriminative information is integrated into a predictive model using ML. As such, the models may provide higher power of discrimination, measured through ROC curves and accuracy [12,14]. These techniques can be particularly useful in processes deemed to be nonlinear in nature.

Methods of feature extraction represent another link in the computational chain of steps involved. The dual-tree complex wavelet transform used in this study provides both time and frequency localization for non-stationary signals. In contrast, FT decomposes a signal into sinusoids that are globally averaged. Therefore, information that is transiently present over a limited period (i.e. time resolution) is lost. Furthermore, the field of non-linear dynamics provides appropriate methods for characterizing chaotic data. QPD-PD method is able to capture the non-linear dynamical nature of VF signal in order to extract features. In contrast, FT is severely limited in its capabilities to properly decompose non-stationary biomedical signals due to its linear and deterministic assumptions, in order to extract the features.

A QVM approach that relies solely upon one ECG feature to predict defibrillation success may suffer from random effects [44]. In contrast to the single feature AMSA technique, the ML approach is able to integrate multiple features in order to construct a more complete/robust model capable of predicting shock success for cardiac arrest victims with greater accuracy. The approach described allows for integration of information from multiple signals, not just multiple features from one signal.

Introduction of other independent but temporally related signals, in this case PetCO_2_, may also help to significantly offset the random effects inherent to ECG features as demonstrated by the increase in sensitivity, specificity, and ROC AUC in the combined signal model. It is not surprising that PetCO2 is helpful in this regard given its relationship to cardiac output and CPP during CPR [45]. Thus, this and other indicators of perfusion may further enhance the performance of an ML based approach to real-time predictive clinical decision-support.

Whenever cross-validation is employed with feature selection or parameter tuning, a twice-nested implementation is requisite for obtaining results that are unbiased by information in the test set. This follows the assumption that field application will produce previously unseen data, providing a true test for the model. Additionally, there is usually a tradeoff between complexity of the predictive model and its generalization performance. As complexity is partly a function of the number of features, type of ML learning algorithm, and its parameters, nested cross-validation also provides a way to optimize this tradeoff.

The definition of ROSC bears mention. While others have utilized alternative definitions that incorporate longer periods of perfusing cardiac rhythm and specific blood pressures, we chose this definition because duration of the post-countershock perfusing rhythm is subject to many confounding variables as well as the potential for an ever-increasing number of post-resuscitation interventions such as therapeutic hypothermia, which has been shown to increase shock success between 12.5% to 50% in vivo [46]. Resuming compressions within a longer post-countershock period (such as 90 seconds for AMSA) confounds the cause of an outcome for decision support model development purposes. On the other hand, not being able to resume CPR for observational sake would be even more problematic. Defibrillation success is also influenced by post-shock pauses, thereby supporting the use of shorter, clinically relevant, definition of ROSC [47,48].

The authors recognize some important limitations to our findings presented here. This analysis was conducted retrospectively upon 358 defibrillation attempts of 153 victims of VF cardiac arrest and our measured outcome, initial ROSC, does not include survival to hospital discharge. Pre-shock pauses and “no-flow” time before defibrillation were not controlled for and have been shown to influence defibrillation success [49]. Additional factors such as certain drugs in the bloodstream, ischemic cardiomyopathy may confound defibrillation success. Cases presenting electromechanical dissociation would not benefit from the proposed model. PetCO2, while shown to dramatically improve the sensitivity and specificity of the model, was not available for all ECG tracings.

## Conclusion

For a given desired sensitivity, MDI provides a significantly higher accuracy and specificity than AMSA in yielding far fewer futile defibrillations (i.e. false positives). Various assumptions underlying feature extraction survive validation through multivariate statistical and non-linear methods. Addition of PetCO_2_ improves the ROC and sensitivity of MDI prediction model. A combined use of appropriate nonlinear modeling techniques, multiple physiologic signals, and machine learning techniques that integrate information from multiple features should facilitate more robust performance when creating predictive physiologic indices for use during cardiac arrest resuscitation.

## Supporting Information

S1 Data Sample dataset with Shock Outcome coded as the last column labeled ‘SO’, and principal component predictive attributes/features represented by the rest of the columns. (CSV)
